# Multi-Dimensional Spectral Single Molecule Localization Microscopy

**DOI:** 10.3389/fbinf.2022.813494

**Published:** 2022-03-04

**Authors:** Corey Butler, G Ezequiel Saraceno, Adel Kechkar, Nathan Bénac, Vincent Studer, Julien P. Dupuis, Laurent Groc, Rémi Galland, Jean-Baptiste Sibarita

**Affiliations:** ^1^ Univ. Bordeaux, CNRS, Interdisciplinary Institute for Neuroscience, IINS, UMR 5297F-33000, F-33000, Bordeaux, France; ^2^ Imagine Optic, Orsay, France; ^3^ Ecole Nationale Supérieure de Biotechnologie, Laboratoire de Bioengineering, Constantine, El Khroub, Algeria

**Keywords:** single molecule localization, single particle tracking, spectral imaging, multi-emitter fitting, live cell imaging

## Abstract

Single molecule localization (SML) and tracking (SPT) techniques, such as (spt)PALM, (u/DNA)PAINT and quantum dot tracking, have given unprecedented insight into the nanoscale molecular organization and dynamics in living cells. They allow monitoring individual proteins with millisecond temporal resolution and high spatial resolution (<30 nm) by precisely localizing the point spread function (PSF) of individual emitters and tracking their position over time. While SPT methods have been extended to study the temporal dynamics and co-organization of multiple proteins, conventional experimental setups are restricted in the number of proteins they can probe simultaneously and usually have to tradeoff between the number of colors, the spatio-temporal resolution, and the field of view. Yet, localizing and tracking several proteins simultaneously at high spatial and temporal resolution within large field of views can provide important biological insights. By employing a dual-objective spectral imaging configuration compatible with live cell imaging combined with dedicated computation tools, we demonstrate simultaneous 3D single particle localization and tracking of multiple distinct species over large field of views to be feasible without compromising spatio-temporal resolution. The dispersive element introduced into the second optical path induces a spectrally dependent displacement, which we used to analytically separate up to five different fluorescent species of single emitters based on their emission spectra. We used commercially available microscope bodies aligned one on top of the other, offering biologists with a very ergonomic and flexible instrument covering a broad range of SMLM applications. Finally, we developed a powerful freely available software, called PALMTracer, which allows to quantitatively assess 3D + t + λ SMLM data. We illustrate the capacity of our approach by performing multi-color 3D DNA-PAINT of fixed samples, and demonstrate simultaneous tracking of multiple receptors in live fibroblast and neuron cultures.

## 1 Introduction

Single Molecule Localization Microscopy (SMLM) relies on the optical- (Betzig et al., 2006; Rust et al., 2006; Heilemann et al., 2008) or binding-induced (Jungmann et al., 2014) spatial isolation and computational localization of individual fluorophores attached to a protein of interest. It provides unprecedented biological insight into the nanoscale organization and dynamics of biomolecules, and has allowed major discoveries in cell biology and neuroscience (Choquet et al., 2021; Lelek et al., 2021). The combined analysis of molecular clustering and diffusive properties, known as SPT, allows to identify the organization of a population of a protein of interest with nanometric resolution and single molecule sensitivity (Cognet et al., 2014; Sibarita, 2014), opening up the potential to infer their interactions with other partner proteins. However, SMLM, as any light microscopy technique, suffers from an inherent trade-off between spatial, temporal and spectral resolutions. The localization accuracy, ie. the precision with which the spatial coordinates of a single emitter can be retrieved, scales with $\sqrt{N}$ , *N* being the number of detected photons above the background per single molecule event (Thompson et al., 2002). It determines the ultimate spatial resolution that can be achieved and defines the minimum displacement that can be distinguished between consecutive frames. As a consequence, collecting and analyzing multicolor 3D SMLM data remains a very challenging problem, where the light collected from single molecules must be allocated to specific dimensions, resulting in an inherent need to sacrifice certain dimensions for others. More specifically, imaging several species with single molecule resolution requires either sacrificing time by imaging each species sequentially, or localization precision by optically slitting the wavelengths using dichroic filters (Friedman et al., 2006; Testa et al., 2010). In live SMLM, sequential imaging is not an option since the goal is usually to monitor simultaneous events, but simultaneously imaging more than two colors in 3D with single molecule resolution is challenging. High-density based single molecule localization approaches can be used to improve temporal resolution (Cox et al., 2012; Zhu et al., 2012; Gustafsson et al., 2016), but at the expense of losing access to single molecular coordinates and therefore single molecule tracking capabilities.

To date, multicolor single molecule fluorescence detection has mostly been implemented using band-pass filters to discriminate between fluorophores (Friedman et al., 2006; Testa et al., 2010). This requires the fluorescence emission spectra of each color to be well separated for minimal signal crosstalk, which limits the number of detectable fluorophores and increases the number of laser lines required to excite all fluorophores. Moreover, splitting the collected photons into several different channels lowers the number of photons per localization event, reducing the localization precision and spatial resolution. Finally, separating the wavelengths with filters requires either splitting and reducing the field of view, or using several cameras for each wavelength, which become complex and expensive for more than two colors. Overall, this strategy compromises the spatial resolution and the acquisition throughput. One alternative is spectral demixing, which allows to use a single excitation line and discriminate up to three spectrally close fluorophores (Lampe et al., 2012; Zhang et al., 2020), but has not been applied for single particle tracking up to date.

Alternatively, a particularly powerful class of approaches aims to distinguish different dyes’ species by directly measuring their spectral signature instead of using emission filters. This is achieved by measuring simultaneously the position and the emission spectrum for each fluorophore. This can be done by combining confocal (Lundquist et al., 2008) or line-scanning excitation schemes (Cutler et al., 2013) with a spectrometer to record the spectral signature. However, confocal-based detection are slow and lack of sensitivity to achieve resolution at the nanometric scale. Another strategy consists in using a diffractive element, such as a prism (Zhang et al., 2015; Moon et al., 2017; Huang et al., 2018; Yan et al., 2018) or a diffraction grating (Bongiovanni et al., 2016; Dong et al., 2016; Liu et al., 2019; Song et al., 2019), in a standard full-field single molecule localization microscope. In these approaches, each fluorophore generates two images: an image without light dispersion to access to the spatial position of the molecules, and a spectral image using a dispersive optical element. Single objective implementations, which split the collected photons in two different detection paths for localization and spectral information, have been proposed but at the cost of a lower localization precision (Bongiovanni et al., 2016; Dong et al., 2016; Moon et al., 2017; Yan et al., 2018). Other groups proposed a 4Pi configuration, i.e., using two objectives positioned on either side of the samples, to preserve the localization precision while accessing the spectral signature of the detected emitters (Zhang et al., 2015; Liu et al., 2019). While these 4Pi configuration detected up to four colors simultaneously on fixed cells using highly overlapping emission spectra fluorophores, their horizontal implementations were not compatible with live cell imaging, which is typically performed in an inverted geometry, preventing powerful single particle tracking to be performed. Huang et al. proposed a single objective implementation combined with an environmental controlled chamber to track up to three different proteins simultaneously on a living cell (Huang et al., 2018), but with a reduced localization precision. All these implementations aim also to probe the full spectra of the molecules detected, imposing strong limitations on the single molecule density per frames to separate those large spectra onto the camera. They therefore limit the acquisition speed and/or statistics achievable, and reduce the probability of the observation of potential meeting of observed molecules, which could be the signature of an interaction in between them.

**FIGURE 1:**
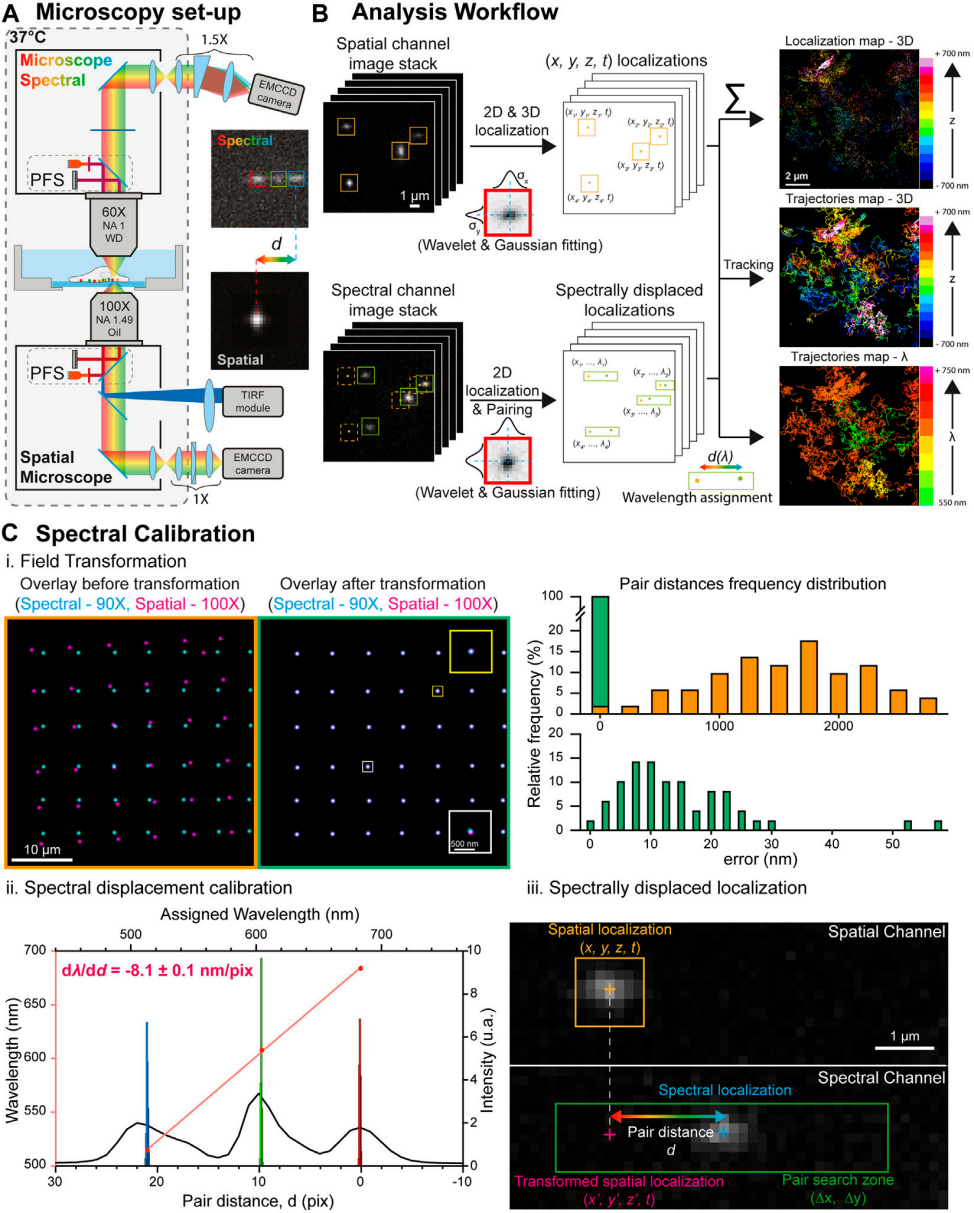
Spectral single molecule microscopy set-up and analysis workflow. **(A)** Microscopy set-up overview: two inverted microscope bodies are positioned one on top of the other to benefit from their stability, automation and active axial feedback through their respective Perfect Focus Systems. The lower (spatial) microscope is equipped with a TIRF/HILO excitation module and a cylindrical lens for astigmatism-based 3D single molecule localization. The upper (spectral) microscope is equipped with a spectral detection arm made of a low dispersive prism (10°) and a ×1.5 magnification relay that converts the spectral information of single molecules into a spatial shift *d*. The insets show 100 nm TetraSpeck beads (excited at 488, 561 and 642 nm) detected by the lower microscope (bottom) and the upper microscope (top) after dispersion, illustrating the spatial shift induced by the prism according to the emission wavelength. **(B)** Spectrally displaced localization workflow. The single molecule localizations in spatial and spectral image stacks are computed using wavelet filtering and bidimensional Gaussian fitting. The localization of the single molecule events in the image stack acquired on the spatial channel enables retrieving their 3D coordinates with nanometric accuracy and reconstruct their trajectories. The spectrally displaced localization, which enables to compute the single molecule’s spectral signature, is performed by pairing the localizations of the same single molecule in both channels and computing their relative distance *d* directly related to the emitted wavelength. **(C)** Spectrally displaced localization calibration. **(i.)** Left: superposition of the localizations of a bead displaced throughout the cameras whole field of view obtained through the spatial (magenta, ×100 magnification) and spectral (cyan, ×90 magnification) channels before (left) and after (right) the field transformation. The insets in the right images shows zooms on two beads to illustrate the field transformation. Right: Histograms of the distances between the paired spatial and spectral localizations before (top) and after (bottom) field transformation. After field transformation, the mean shift in between the paired localization is below 12 nm. **(ii.)** Calibration of the prism-induced spectral displacement: The black curve represents a line scan along the three emission peaks of a TetraSpeck bead detected on the spectral channel. Their individual localizations on 1,000 successive frames (histograms) enabled to compute their respective spatial shift depending on their central wavelength (red dots). Linear regression (red curve) leads to the calibration of the prism-induced spatial shift according to the emitted wavelength used for spectrally displaced localization. **(iii.)** Principle of the spectrally displaced localization process: The localization of a single molecule on the spatial channel is reported in the spectral channel after field transformation. A pair search zone (green rectangle) defined according to the prism dispersion axis and an *a-priori* knowledge of the molecule wavelength enables to pair it with its localization on the spectral channel. The pair distance allows the precise determination of the emission wavelength to the localized molecule.

We here detail an acquisition and analysis framework for versatile multidimensional (3D + t + λ) SMLM (Figures 1A,B), with a focus on its capability to perform 3D multicolor single particle tracking using a spectral detection configuration. Composed of commercially available standard equipment and software, and a freely available analysis solution, we demonstrate that optimal spectral SMLM (up to five colors) can be performed without compromising the 2D and 3D localization and tracking performance. We illustrate 3D multicolor imaging of various fixed and living samples, including the simultaneous nanoscale monitoring of neuronal synaptic receptors’ dynamics. We show that using both the spatial and the spectral information we can achieve optimal multidimensional single molecule localization.

## 2 Materials and Methods

### 2.1 Microscopy Setup

We devised a custom spectral microscope with a 4Pi configuration for versatile (3D + *t* + *λ*) SMLM. It is composed of two commercially inverted microscope bodies (Nikon TiE) precisely aligned one on top of the other: a first microscope, placed below, to perform state of the art (3D + *t*) SMLM (called direct or spatial), equipped with an azimuthal TIRF/HiLo illumination device (iLAS2, Gataca Systems); a second microscope, placed on top, for spectral (*λ*) characterization using photons usually lost in traditional mono objective configurations (Figure 1A, Supplementary Figure S1). The two microscope bodies are precisely aligned by translating the bottom microscope using a (*x, y, θ, φ*) stage placed below the bottom microscope (UMS, Scientifica). Such a geometry allows i) to perform state of the art 3D localization using all the photons collected by one high NA TIRF objective (×100 Oil NA1.49, Nikon); ii) to determine the spectral signature of the detected fluorophores using a second high NA objective (×60, Water Dipping NA1, Nikon), without compromising the localization performances (Figure 1B). The choice of using commercial microscopes is to benefit from existing hardware (filter cube, objective turret, TIRF illumination module, Perfect Focus System), motorization, software control, and stability for long-term acquisitions. It also provides a user-friendly environment that biologists are familiar with. The whole 4Pi microscope is caged in a custom plexiglass enclosure heated at 37°C for live cell experiments (Life Imaging System) and driven by the MetaMorph software (Molecular Devices). The conventional vertical architecture enables using standard live sample holders, simplifying live sample preparation protocols and imaging. Two synchronized sensitive EMCCDs (Photometrics EVOLVE 512B), one for each detection path, allow the microscope to track bright quantum dots as well as dimmer organic fluorophores and fluorescent proteins across the entire field of view of the EMCCDs, nearly 80 µm × 80 µm @ 30Hz, using conventional filter sets and dichroic mirrors. Axial stability, a crucial parameter for maintaining the desired focal planes for each imaging path, is ensured by their respective integrated Nikon Perfect Focus System (PFS), allowing real-time axial drift compensation based on LED reflection at the coverslip surface. We used special custom filters to ensure the two PFS systems to be used simultaneously without interfering (Supplementary Figure S2). Before PFS activation, both spatial and spectral imaging planes are precisely adjusted independently to overlap to each other using fiduciary markers adsorbed at the glass coverslip, and this focal plane overlap is maintained post-PFS activation using the PFS’s axial offset.

**FIGURE 2:**
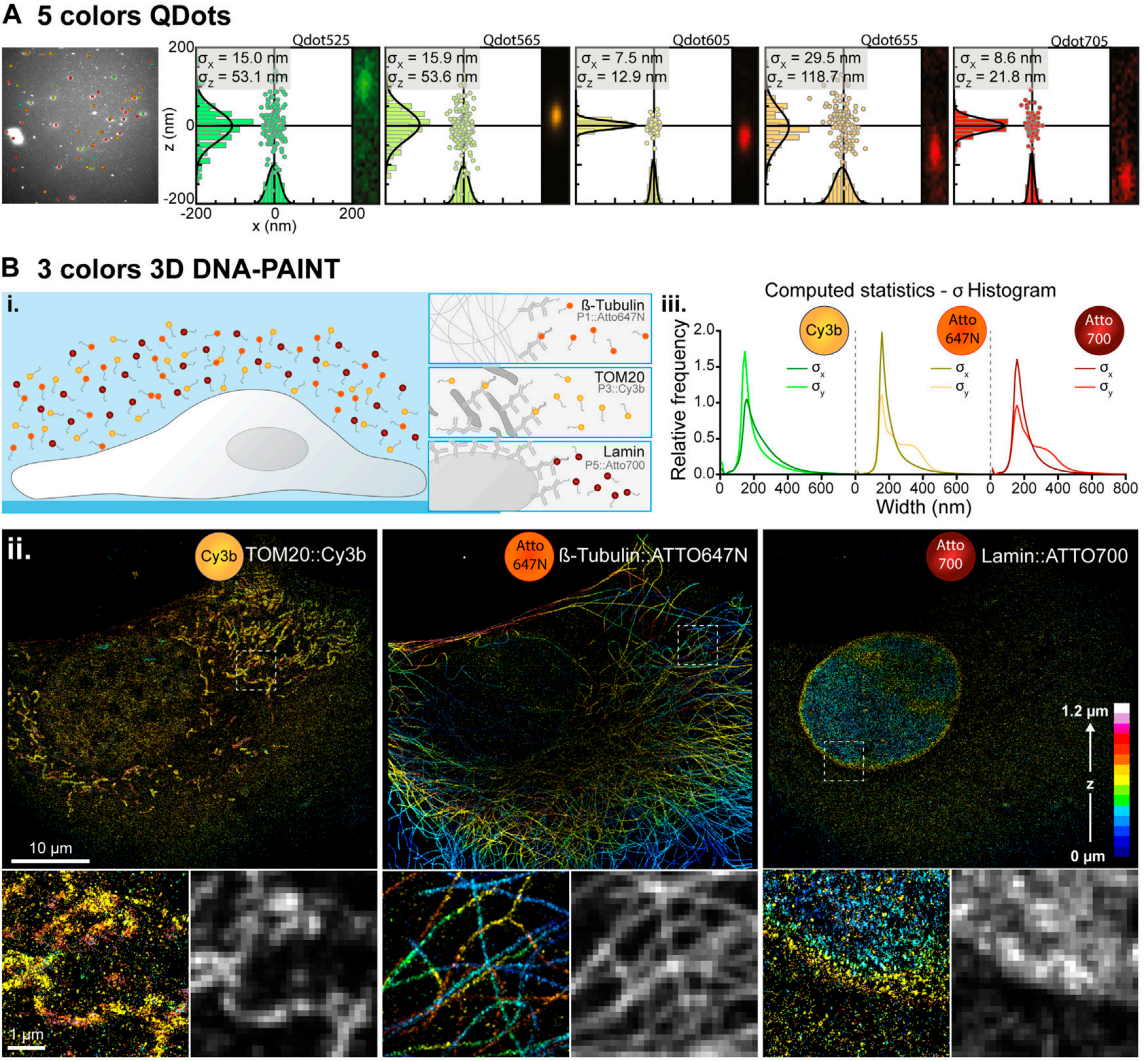
Simultaneous multi-color single molecule detection. **(A)** Simultaneous detection of five spectrally different Qdots. Left: Image of a field of view containing five spectrally different populations of Qdots with their overlaid localization and emission wavelength determined by spectrally displaced localization. Right: Spectral localizations of Qdots computed from 1,000 consecutive frames for each population of QDots with their localization histogram along the x- and z-axis and the associated Gaussian fitting (black curves) for pointing accuracy estimation. The insets represent the images of those Qdots in the spectral (upper) channel inside the pair search zone. **(B)** Simultaneous three colors 3D DNA-PAINT imaging. **(i.)** Experiment principle: Three biological structures of interest (microtubule, TOM20 and LaminB1) are labelled within a COS-7 cell with orthogonal DNA docking strands each associated with an imager strand conjugated to a different fluorescent dye (Atto647N, Cy3b and Atto700 respectively). **(ii.)** Depth color-coded super-resolved 3D reconstructions of the three structures of interest acquired simultaneously and filtered in wavelength to extract each of the three dyes populations. Intensity images have been reconstructed using the ImageJ ThunderSTORM plugin to create a blur effect according to the single molecule localization precision. The lower panels represent zoomed-in images of the dotted white boxes and their comparison with diffraction-limited images of the same areas reconstructed using a fixed Gaussian blur of 100 nm (resp. 250 nm) standard deviation in XY (resp. Z), to simulate diffraction limited spatial resolutions of 230 nm (resp. 575 nm). **(iii.)** Histograms of the astigmatism-based PSF widths along the x- ( $\sigma_{x}$ ) and y-axis ( $\sigma_{y}$ ) for each dyes’ population computed by bidimensional Gaussian fitting.

**FIGURE 3:**
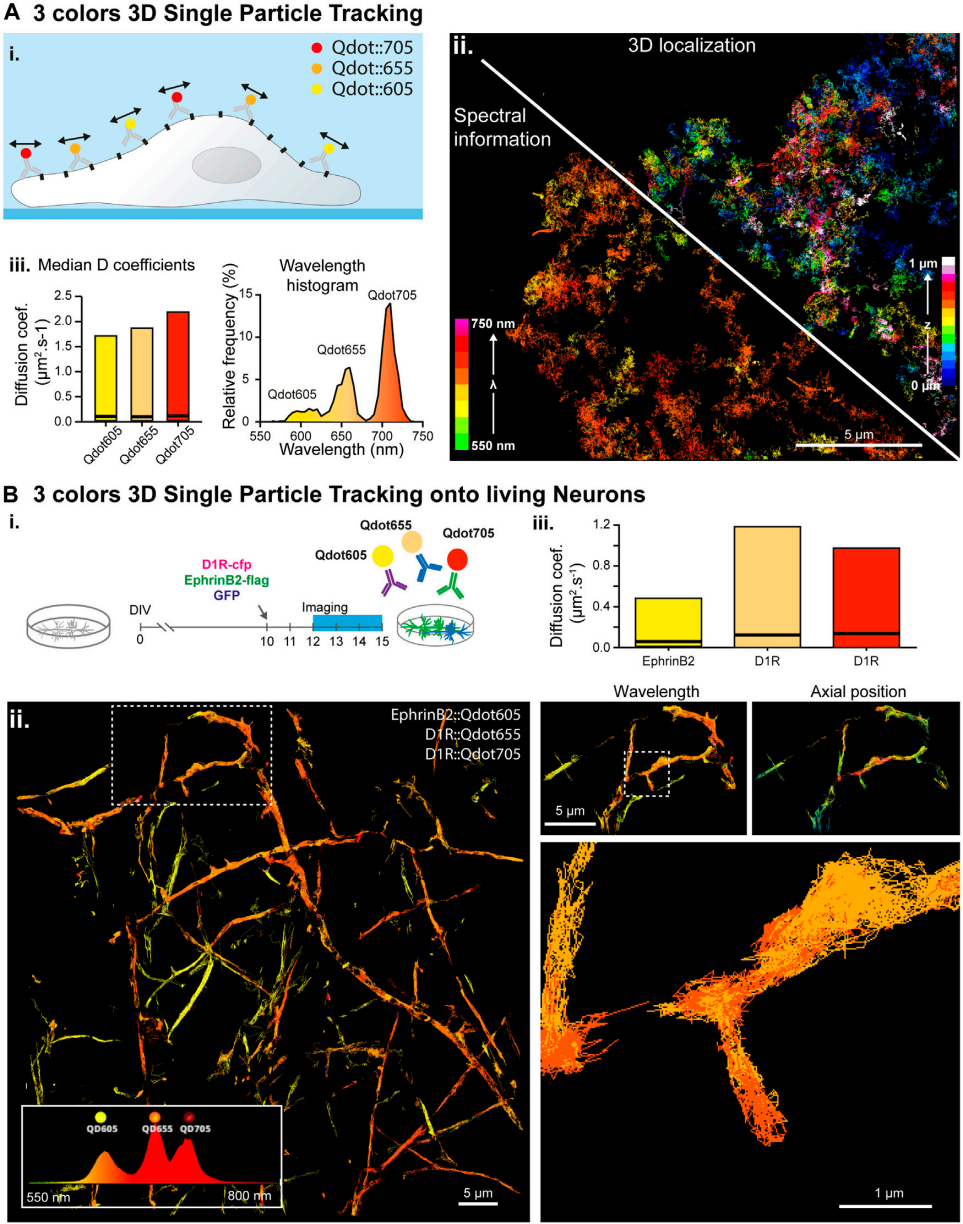
Simultaneous multi-color 3D single molecule tracking. **(A)** Simultaneous three colors 3D single particle tracking on living COS-7 cells. **(i.)** Experiment principle: NCAM membrane receptors in a COS cell are labelled with three populations of spectrally different QDots and tracked in 3D over time. **(ii.)** Reconstructed trajectories of the detected Qdots color-coded for their depth (upper right corner) or for their assigned wavelength (bottom left corner). **(iii.)** Computed median and range of diffusion coefficients for each QDots population (left) and histogram of the wavelengths detected during the acquisition revealing the three Qdot populations (right). **(B)** Simultaneous three colors 3D single particle tracking on living neurons. **(i.)** Experiment principle: Hippocampal dissociated neurons culture are transfected at DIV-10 with the plasmids coding for D1R-cfp, EphrinD2-Flag and soluble GFP. In between DIV12 and DIV15, expressed D1R-cfp proteins are labelled with two populations of spectrally different Qdots (Qdot 655 and Qdots705) and expressed EphrinB2-flag proteins are labelled with Qdots605 before being imaged and analyzed by spectrally informed localization and tracking. **(ii.)** Left: Reconstructed trajectories of the three Qdots populations. Right: Successive zooms of the white dotted regions revealing the main and well distinct zones explored by the two receptors (top) and the trajectory of two spectrally different Qdots (Qd655 and Qd705) within a spine (bottom). **(iii.)** Median and range of diffusion coefficients for each of the three different Qdots population, revealing that the two Qdots conjugated to the same receptors (D1R) behave similarly whereas EphrinB2 receptors diffuse slower.

On the lower detection path, we use an astigmatism-based approach (manual N-STORM kit, Nikon) to achieve state of the art 3D localization (Figure 2) and tracking (Figure 3). On the upper spectral detection path, we use a low dispersive prism (10° edge prism, PS814-A, Thorlabs) placed in the Fourier plane of a 4f imaging relay to access to the spectral signature of the detected emitters. This 4f system integrates a ∼×1.5 zoom to optically match the spatial and spectral FOVs as closely as possible, with a magnification of ×100 and ×90 respectively. Such a relatively low diffracting prism, compared to the highly dispersive element commonly used in other hyperspectral systems so far (Zhang et al., 2015; Bongiovanni et al., 2016; Dong et al., 2016; Moon et al., 2017; Huang et al., 2018; Yan et al., 2018; Song et al., 2019), enables computing the colour of the single emitters without spreading their whole emission spectra onto the upper camera. Hence, it allows higher single molecule density experiments to be performed, which is particularly important to monitor statistically rare events. This dispersive element converts each emitter’s wavelength into a spatial displacement, laterally shifting the localization of the single emitter linearly with respect to its spectral mean. We measured a spectral dispersion of −8.1±0.1 nm/pixel using multicolor diffraction limited microbeads (100 nm) with well-defined fluorescence spectra. Once calibrated, the emission wavelength of each localized molecule is precisely determined by computing the dispersion-induced spatial displacement, thanks to the localizations pairing between the upper and the lower detection paths (see below) (Figure 1C). Critically, the low dispersion contains the spectrum of common fluorescent dyes to just a few pixels, which allows the use of conventional localization algorithms.

By building on top of commercial microscope bodies, we were able to modify various components to optimize and streamline the usability of the microscope, most notably with the objective configurations. While the original implementation used matching 100X NA 1.49 oil objectives on the spatial and spectral paths, we ran into several difficulties that limited routine use on living samples in this configuration. Since our system supports mismatched objectives, we chose to use a 60X NA1 water dipping objective (90X after 1.5X additional magnification) on the upper optical path, with a 100X NA1.49 oil immersion objective on the lower path, thereby avoiding several optical and mechanical limitations of the matched objective configuration. Mismatching the objectives allowed us: (1) to maintain the collection efficiency of oil immersion objectives for 3D localization and the capacity to perform TIRF illumination, (2) to simplify the sample mounting without the need to mount samples in between two closely spaced coverslips (≈30 µm), (3) to increase the field of view (by 10%) and the depth of field (from ≈0.4 to 0.7 µm) of the spectral channel, and (4) to reduce spherical aberrations induced by imaging 30 µm deep through a coverslip with a high NA oil immersion objective. Altogether, the use of a 60X NA1 water dipping objective on the spectral channel greatly reduces the experimental complexity of using the system by allowing the use of conventional, open-top sample holders and maximizing the probability that the upper channel will collect spectral data for each localization on the spatial channel.

### 2.2 Single Molecules Localization Analysis

We have developed a complete analysis software solution, called PALMTracer, to analyze and represent multi-dimensional (*x, y, z, t, λ*) SMLM data. It is developed as a plugin of MetaMorph software, to be used either directly on the microscope during the acquisition and provide rapid user-feedback, or in off-line mode to analyze the data post-acquisition. It allows standard 2D and 3D single molecule localization and tracking as well as advanced spectral analysis, integrating quantitative analysis of diffusive properties with various visualizations. It is the result of 10 years of developments with biologist end-users (Rossier et al., 2012; Nair et al., 2013; Garcia et al., 2015; Chamma et al., 2016; Beghin et al., 2017; Floderer et al., 2018; Jullié et al., 2020; Compans et al., 2021), and comes with an intuitive graphical user interface (GUI) (Supplementary Figure S3). It integrates a powerful batch engine enabling to systematically and sequentially analyze several files in an entirely automatic fashion, greatly speeding up the analysis of various experiments. Many filtering options are available on the various statistics that can be computed from SMLM localization (Gaussian fit properties) and tracking analysis (diffusion properties) as well as on the spectral characterization.

#### 2.2.1 Single Molecule Localization and Tracking

Single molecule localization is performed using a combination of wavelet decomposition and Gaussian fitting (Izeddin et al., 2012; Kechkar et al., 2013). Wavelet decomposition allows detection of isolated single emitters quickly and robustly to noise and background (Izeddin et al., 2012), while Gaussian fitting enables the precise coordinates determination of the detected emitters, in two and three dimensions (Kechkar et al., 2013). Once localized, molecule trajectories are computed from the molecular coordinates using a simulated annealing algorithm (Racine et al., 2006). Diffusion coefficients are extracted from the trajectories by linear fitting the Mean Square Displacement (MSD) curves, which represent the surface, or volume in 3D, explored by a molecule over time (Sibarita, 2014). Localization coordinates and trajectories, and their related quantitative features (e.g., Gaussian fitting parameters, MSD, diffusion coefficients, etc … ) are saved in various CSV files, compatible with popular analysis and visualization software such as VISP (El Beheiry and Dahan, 2013), ThunderSTORM (Ovesný et al., 2014) or SR-TESSELER (Levet et al., 2015) for advanced rendering and point-cloud analysis.

#### 2.2.2 Spectral Analysis by Spectrally Displaced Localization

The spectral determination of each localized molecule is achieved by spectrally displaced localization. It consists in pairing the localizations of single emitters obtained from the two images of the same focal plane collected simultaneously from the direct (lower) and spectral (upper) detection paths, and measuring the spatial shift induced by the prism in the upper detection path (Figure 1C–iii). Indeed, this dispersive element converts the emitter wavelength (*λ*) into a spatial displacement (*d*), by shifting in a first approximation the localization linearly as a function of the emission wavelength. This spatial shift allows to easily retrieve the emission wavelength of the emitters localized in the lower detection path, allowing their accurate (*x, y, z, t, λ*) determination.

Spectrally displaced localization is computationally simple and intuitive, as it only relies on standard localization and pairing algorithms. It is a three-step process:

(1) Superposition of the spatial and spectral channels: a calibration step is required to compensate for differences in magnification, rotation, or other field of view distortions between the direct (lower) and spectral (upper) detection paths. The field of view transformation is computed from raster scanning images of a fiducial marker having a single fluorescence emission peak (fluorescent nanodiamonds excited at 647 nm in our case) in a 7 × 7 grid, covering the whole field of view of the two detection paths (Figure 1C–i). The corresponding 49 centroids positions are first localized in each channel and paired by nearest neighbor search. Then, a two-dimensional 3^rd^ order polynomial transformation is computed from the paired coordinates by least-squares Levenberg-Marquardt minimization algorithm:
$$x^{\prime }=a_{x,1}x^{3}+a_{x,2}y^{3}+a_{x,3}x^{2}y+a_{x,4}xy^{2}+a_{x,5}x^{2}+a_{x,6}y^{2}+a_{x,7}xy+a_{x,8}x+a_{x,9}y+a_{x,10}$$ (1)
$$y^{\prime }=a_{y,1}x^{3}+a_{y,2}y^{3}+a_{y,3}x^{2}y+a_{y,4}xy^{2}+a_{y,5}x^{2}+a_{y,6}y^{2}+a_{y,7}xy+a_{y,8}x+a_{y,9}y+a_{y,10\;}$$ (2)
  where *x′* and *y′* are the transformed coordinates of *x* and *y*, respectively with coefficients *a*_*x,n*_ and *a*_*y,n*_ *.* This 3^rd^ order polynomial transformation requiring 10 coefficients per spatial dimension is used to render the system as versatile as possible and correct for any non-linear field of view deformations induced by the spectral optics or the cylindrical lens. Once calibrated, this transformation is applied to the lower localizations to match the field of view of the upper camera, resulting in a field-dependent error after transformation typically ranging from 0 to ∼60nm, with median values around ε = 12 nm (Figure 1C–i). Due to the prism in the spectral detection path, this field of view transformation is wavelength dependent, and this alignment process creates a transformation centered at the spectral mean wavelength $\lambda_{0}$ of the emission of the fiducial marker used.
(2) Pairing of the detected molecules on both channels: Once the direct (lower) coordinates are transformed to match the spectral (upper) coordinates, a linear search around the transformed lower localization is performed to find its paired upper localization (Figure 1C–iii). The displacement angle of the prism is aligned with the EMCCD chip to induce a displacement of the localizations in one axis direction as a function of the wavelength (*y*-axis in our case). It allows minimizing the pair search zone to a reduced linear zone. Minimizing the size of this pair search zone is important as it defines the maximum density of simultaneously fluorescing single molecules per image frame, minimizing missed pairing. The width ( Δx ) and height ( Δy ) of the search zone are both user-definable based on *a priori* knowledge of the fluorescent species being imaged. Once the localizations are matched, their pair distance, d , is calculated as $d=y_{upper-}y{'}_{lower}\;$ and retained as a proxy for the spectral mean of the emitter.
(3) Wavelength determination: The wavelength ( λ ) of each paired localization is assigned upon spectral calibration using the following linear function: $\lambda =\lambda_{0}+\alpha d$ , $\lambda_{0}$ being the reference emission wavelength and α the calibration spectral coefficient. The spectral calibration is performed by imaging isolated tetraspeck multicolor beads (N = 7 beads, 100 planes) adsorbed on a glass coverslip, exciting at λ_exc_ = 488 nm (blue), λ_exc_ = 561 nm (orange), and λ_exc_ = 640 nm (red). The system was aligned using the red fluorophore as a reference ( $\lambda_{0}=683\;nm$ ) and a distance of 20.90±0.06 pixels (resp. 9.69±0.04 ) was measured with the blue (resp. orange) fluorophores. Distances were computed from the localization of the three detected peaks of the tetraspeck beads. We considered the mean emission wavelength integrated over the quad-band filter used in detection ( $\lambda_{red}=683\;nm;\;\lambda_{orange}=607\;nm;\;\lambda_{blue}=514\;nm$ ). This calibration allows the precise determination of the spectral dispersion coefficient α=−8.1±0.1 nm/Pix of our spectral detection arm (Figure 1C–ii).

It is important to note that the spectrally displaced localization process does not alter the accuracy of the spatial localizations from the lower detection path, and merely provides additional spectral information for each localization with no photon cost. In addition, the spatial localization of the emitter’s signature in the spectral (upper) channel can also provide additional spatial information allowing overlapping spectrally different emitters to be separated by spectrally-informed multi-Gaussian fitting (Section 2.2.4). However, in this specific case of spatially overlapping fluorophores, spectrally displaced localization efficiency is limited by their spectral proximity to several tens of nanometers, depending on the prism-induced spectral dispersion and the spectral width of the fluorophores’ emission. This therefore limits the maximum number of fluorophores that can be readily monitored simultaneously.

#### 2.2.3 Lateral Drift Correction

Lateral drifts in either the lower or upper detection path affect the pair distance between matched localizations, deteriorating the spectrally displaced localization quality during the acquisition (Supplementary Figure S4). To avoid time-dependence in the spectral assignment, it is necessary to numerically compensate for the drift on each path post-acquisition. We used fiduciary markers to compute and compensate for lateral drifts on each channel separately to correct for drifts incurred during the image acquisition process. Additionally, lateral shifts between the fields of view accumulated between acquisitions on the same coverslip were corrected by simply updating the zero-order coefficients of the field of view transformation ( $a_{x,10}$ and $a_{y,10}$ of Eqs 1, 2), allowing the use of a previously computed field transformation while compensating for spatial drifts. These automatic corrections of the FOV transformation and independent channel drifts ensure that the two channels remain aligned (pair distance = 0) at a fixed wavelength as long as the same fiducial markers and filter sets are used.

#### 2.2.4 Spectrally-Informed Multi-Gaussian Fitting

**FIGURE 4:**
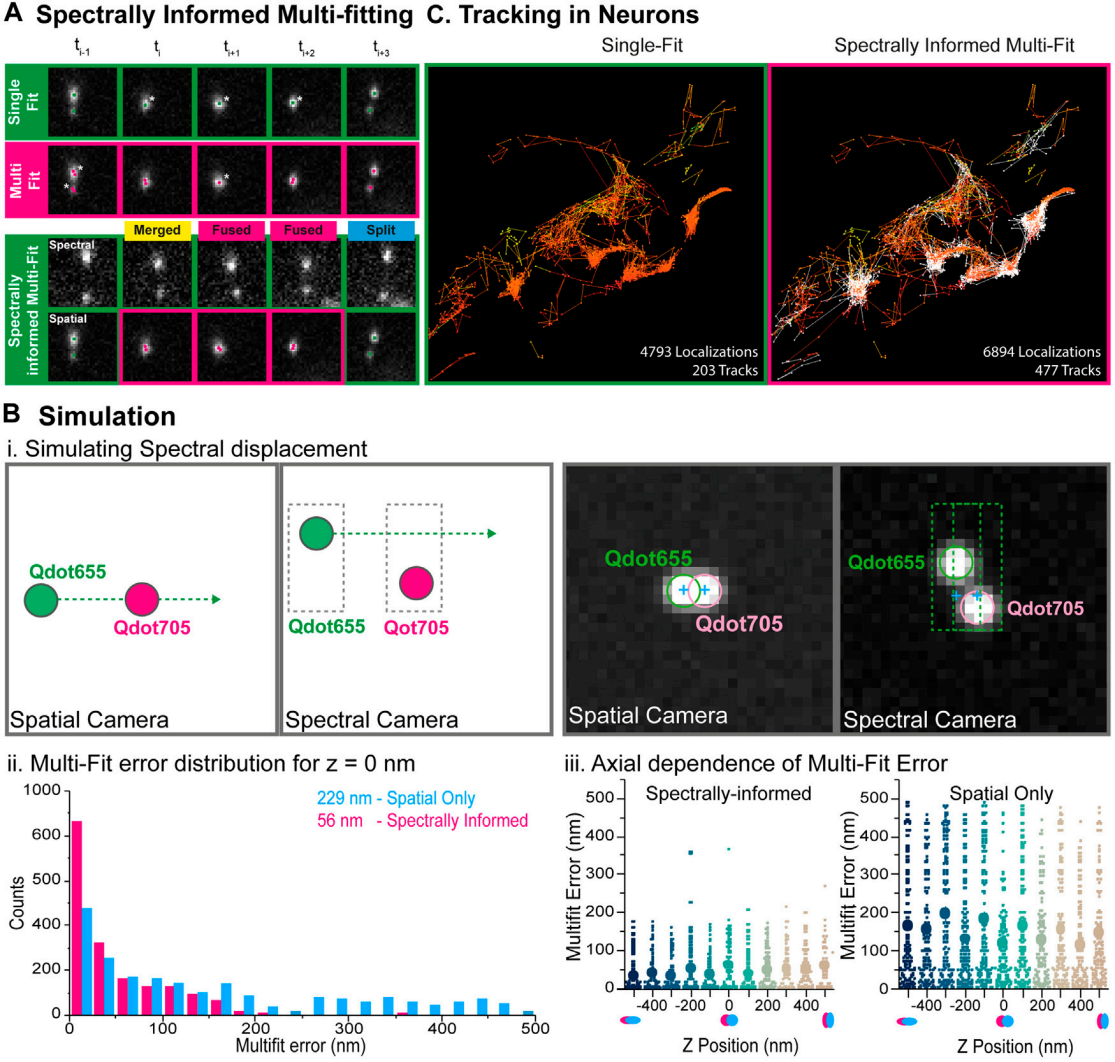
Spectrally-informed multi-fitting principle and benchmarking through simulations. **(A)** Principle of the spectrally-informed multi-Gaussian fitting method. When two fluorescent single particles diffuse together within an area smaller than the diffraction limit of light, it becomes impossible to localize them individually and extract their exact trajectories. A systematic single Gaussian-fitting approach leads to under-counting localization (top) whereas a systematic multi-Gaussian fitting approach sometime leads to over-counting localizations (middle) (Stars indicate over- or under-counting errors in the localization process). If those particles have different spectral signatures, they will be separated in the spectral channel, allowing to precisely determine the number of particles, and to use the multi-Gaussian fitting algorithm when needed to retrieve their coordinates (bottom). The Spectrally-informed multi-Gaussian fitting (magenta) occurs when two emitters overlap on the spatial channel (single localization) and two distinct localizations are detected on the spectral channel. **(B)** Multi-Gaussian fitting simulation for benchmarking. **(i.)** Simulation principle: Two spectrally-distinct emitters (QDot 655 and QDot 705) were simulated for the spectral and spatial cameras for 320 frames. QD655 displaces horizontally across the field of view at 10nm/frame such that the emitters begin separated, then overlapped on the spatial camera but not on the spectral camera, and finally separated again. Right: True camera noise and PSF sizes were used in the simulations. The two emitters overlap on the spatial camera, but not on the spectral camera. **(ii.)** The localization error for each of the multi-Gaussian fitting methods (without and with spectral information) is calculated and compared to the simulated localization for simulated separation distances up to +/- 500 nm. Systematically performing the multi-Gaussian fitting results in a mean square error of 229 nm (magenta), while utilizing the spectrally-informed multi-Gaussian fitting algorithm results in a 4x reduction of localization error to 56 nm (cyan). **(iii.)** The same horizontal-displacement simulation was then performed for various simulated astigmatic z-positions between −500 and 500 nm. Mean error for each of z-position is plotted for each fitting method (without (right) and with (left) spectral information), illustrating that the spectrally-informed multi-fitting significantly improves the localization precision across the range of assignable axial positions. **(C)** Simultaneous three colors Qdots tracking within living neurons. Reconstructed trajectories computed from systematic single-emitter fitting (left) or spectrally informed multi-Gaussian fitting (right). Spectrally-informed Gaussian fitting leads to ≈1.5 times more localizations and ≈2 times more trajectories.

In order to distinguish overlapping single molecule signals, occurring when single emitters are separated by less than ∼200 nm one from each other, a common solution is to use multi-Gaussian fitting algorithms (Holden et al., 2011; Huang et al., 2011; Babcock et al., 2012). However, in absence of knowledge of the number of single molecule candidates (*n*), systematic multi-gaussian fitting can lead to artefacts, especially for astigmatism-based 3D localization, which requires more parameters to estimate (Sage et al., 2019). Here, we take advantage of the localization information in the spectral channel to determine the number of molecules to localize, and select either a single or a multiple Gaussian fitting algorithm (Figure 4A). Such spectrally-informed multi-Gaussian fitting enables to (1) constrain the number of emitters that have to be retrieved and (2) initialize the spatial positions of those emitters perpendicular to the prism dispersion axis in the fitting process. This is of course only possible if the overlapping emitters have emission wavelengths sufficiently different that they result in two distinct localizations on the spectral channel. The number of single molecule candidates to localize by spectrally-informed multi-Gaussian fitting, either one or two, is determined by the number of localizations in the pairing area ( Δx , Δy ) of the spectral image (Section 2.2.2 for definition). We further exploit the information from the spectral channel to improve the accuracy of the multi-Gaussian fitting algorithm by initializing it with the spatial localization in the dimension perpendicular to the spectral displacement, ie. with the x-coordinates if the spectral dispersion is in the y-direction. Such a capacity allows monitoring several proteins simultaneously exploring the same nanoscopic environment smaller than the diffraction limit, opening new venues to monitor potential molecular interactions.

#### 2.2.5 Simulations

The systematic use of a single- or a multi-emitter fitting process may lead to under- or over-counting errors. Indeed, single emitter localization cannot distinguish several emitters within a diffraction limited area, whereas multi-emitter localization sometime detects several emitters in a region where there is only a single emitter (Figure 4A). In order to characterize and validate the spectrally-informed multi-Gaussian fitting algorithm, we simulated dual-camera SMLM acquisitions using real-world conditions (Figure 4B). We first generated a “ground-truth” localization file containing two spectrally distinct emitters (Qdot655 and Qdot705) on the spatial and spectral images for 320 frames. The intensity and spectral dispersion of the emitters were assigned based on real acquisition data to ensure similar spectrally displaced localization precision. Second, this ground-truth localization file was used to generate image stacks with simulated PSFs. Per-pixel noise statistics were analyzed from real acquisitions and used as background, again to ensure a localization precision similar to real-life conditions. Qdot705 was positioned statically at the center of the frame and Qdot655 displaced horizontally across the field of view at 10 nm/frame, such that the emitters begin separated, then overlap on the spatial image, but never on the spectral image, and then separate again (Figure 4B–i). Finally, these simulated image stacks were then analyzed using only the spatial information by single- and multi-fitting algorithms, as well as the spectrally-informed analysis, and compared with the ground-truth localizations. We observed that, when only the spatial information is used, conventional multi-Gaussian fitting resulted in a mean fit error of 229 nm (Figure 4B–ii). Such a large mean error stems mainly from pairing errors due to over-localization obtained by systematic multi-Gaussian fitting. Analyzing the same dataset using the spectrally-informed multi-Gaussian fitting resulted a mean fit error of 56 nm, a 4-times improvement that enables more accurate SPT of two spatially overlapping emitters (Figure 4B–ii). This difference is explained by the elongation of the astigmatism-based point-spread function with respect to the axial position of the emitters (Figure 4B–iii).

### 2.3 Imaging Experiments

#### 2.3.1 Simultaneous Detection of Five Qdots

Five different populations of streptavidin conjugated Qdots (Qdot525, Qdot565, Qdot605, Qdot655 and Qdot705, Q10151MP, Invitrogen) were physically adsorbed onto an 18 mm clean coverslips at a very low concentration (Figure 2A). The exact concentration for each population was first sequentially adjusted. A solution of a unique diluted streptavidin labelled Qdot population was incubated for 10 min on a clean 18 mm coverslips along with 100 nm fluorescent nanodiamond acting as fiduciaries and reference for spectral calibration (1:1,000 from stock solution, NDNV100 nmMd10ml, Adamas Nanotechnologies INC.) and then rinsed three times with PBS. The coverslips were then imaged on our spectral microscope in order to determine the density of adsorbed streptavidin labelled Qdots and the concentration adjusted accordingly to ensure single molecule detection regime when mixed with four other Qdots populations. Finally, five population of streptavidin Qdots were incubated for 10 min on a coverslip (concentration: Qdot525: 1:1.10^6^; Qdot565: 1:2.10^6^; Qdot605: 1:5.10^6^; Qdot655: 1:2.10^6^; Qdot705: 1:2.10^6^) along with nanodiamonds, rinsed three times with PBS and mounted onto a Ludin imaging chamber (Life Imaging System). For imaging, all Qdots populations were excited at 488 nm through a Quad-band filter (F66-04TN, AHF) in the lower (spatial) microscope, and a triple laser lines rejection filter (ZET 405/488/561, F67-408, AHF) was added into the upper (spectral) detection path to reject excitation laser light.

#### 2.3.2 Three Color DNA-PAINT Experiments

Cos-7 cells were plated onto 18 mm 1.5H coverslips at 50,000 cells per coverslips for isolated cell experiments. After spreading (around 4 h), cells were prepared using a fixation step with 4% formaldehyde +0.1% Triton X-100 for 10 min, followed by the quenching of the autofluorescence using 150 mM Glycine (Sigma) in PBS for 10 min, and an additional permeabilization step with 0.3% Triton X-100 for 10 min. Unspecific binding sites were then blocked using 3% BSA (Sigma) in PBS for 2 h at room temperature before primary antibodies incubation overnight at 4°C in the same blocking solution. Rat anti-alpha-tubulin (MA1-80017, ThermoFisher) diluted at 1:300, goat anti-Lamin B1 (sc-6217, Santa Cruz) diluted at 1:300 and Rabbit anti-TOM20 (sc-11415, Santa Cruz) diluted at 1:600 were used. After three washing steps with the blocking solution, cells were incubated with the following DNA-PAINT secondary antibodies kindly provided by Ralf Jungmann’s lab for 2 h at room temperature: anti-rat conjugated with the P1 DNA handle (1:100), anti-rabbit conjugated with the P3 DNA handle (1:100) and anti-goat conjugated with the P5 DNA handle (1:100). Cells were finally washed three times with the blocking solution and three times with PBS before being stored at 4°C until imaging. Just before imaging, coverslips were incubated with 100 nm nanodiamonds as fiduciaries at 1:1,000 from the stock solution for 10 min and then rinsed three times with PBS. For imaging, a solution containing the following imager strands (Eurofins) were prepared in an imaging solution (PBS +500 mM NaCl): P1*-Atto647N diluted at 30 pM, P3*-Cy3b diluted at 300 pM and P5*-Atto700 diluted at 200 pM and poured onto a coverslip mounted into a Ludin imaging chamber (the star indicates the complementary of the DNA strand). Concentrations of the imager strands were first adjusted individually for each color to ensure adequate single molecule density before being combined for the simultaneous 3-colors experiment. For acquisition, two laser lines at 561 and 642 nm were simultaneously used to excite the fluorophore Cy3b and Atto647N/Atto700 respectively thanks to a quad band filter set in the lower (spatial) microscope. In the upper detection arm (spectral microscope) a quad-band notch filter 400-410/488/561/631-640 (F40-072, AHF) was added to filter out excitation laser light. 120,000 frames were acquired at 5 Hz and then analyzed with PALMTracer (Figure 2B).

#### 2.3.3 Multiple 3D QDots Tracking of Membrane Proteins on Living Fibroblasts

Cos-7 cells were electroporated with plasmids coding for the protein NCAM:AP and the enzyme, BirA^ER^ according to the manufacturer’s protocol (Lonza), using 2.5 µg (resp. 2 µg) of DNA for 2 million cells. Electroporated cells were immediately seeded on 18 mm 1.5H coverslips at 50,000 cells per coverslips and incubated in high-glucose Dulbecco’s modified Eagle’s medium (DMEM, Sigma) supplemented with 10% Fetal Bovine Serum (FBS, 16000-044, ThermoFisher), 1% GlutaMAX (35050-061, Gibco) and 1% penicillin-streptomycin (Sigma) and with the addition of 10 µM of biotin according the protocol described in Chamma et al. (Chamma et al., 2017). 48 h post-electroporation, cells were incubated with 100 nm nanodiamonds as fiduciaries at 1:1,000 from the stock solution and streptavidin labelled with Qdot605, Qdot655 and Qdot705 diluted at 1:40,000, 1:40,000 and 1:50,000 from stock solution respectively for 10 min. After washing excess of Qdots’ labelled streptavidin with culture media, imaging medium (Fluorobright A1896702, ThermoFisher) supplemented with 10% FBS, 1% Glutamax, and 100 mM Hepes was poured onto the cells for imaging. During imaging a single laser line at 561 nm was used to excite all three Qdot populations through a quad-band filter set in the lower (spatial) microscope, and a triple laser lines rejection filter (ZET 405/488/561) in the upper (spectral) detection path to filter out laser excitation light. Imaging was performed at frame rates ranging from 20 to 100 Hz on field of views ranging from 80 × 80 µm to 20 × 20 µm respectively (Figure 3A).

#### 2.3.4 Multiple 3D QDot Tracking of Synaptic Receptors on Living Neurons

Mixed hippocampal cultures containing both neurons and glial cells were prepared from embryonic stage (E18) Sprague-Dawley rats and maintained in Neurobasal Plus medium (Gibco, A3585911) supplemented with GlutaMAX™ (Gibco, #35050-038), B-27™ Plus (Gibco, A3653401), and 10% horse serum for 3–5 days *in vitro* (DIV), at which time the medium was changed to a horse-serum free B27 Plus-containing Neurobasal Plus medium. Neurons were transfected between DIV-7 and DIV-10 with plasmids encoding D1R-cfp and EphrinB2-flag as well as soluble GFP using the calcium-phosphate coprecipitation method. In between DIV-12 and DIV-14, neurons were then prepared for imaging. They were first incubated for 10 min with a mix of rabbit anti-GFP (#A-6455, Thermo Fisher Scientific Inc., 1:10,000) and mouse anti-Flag (#F-1804, Thermo Fisher Scientific Inc., 1:1,000) primary antibodies, then washed and incubated for 10 min with F (ab')2-Goat anti-Rabbit IgG-coupled Qdot655 (#Q11422MP, ThermoFisher Scientific Inc., 1:50000) or Qdot705 (#Q11461MP, ThermoFisher Scientific Inc., 1:50000), F (ab')2-Goat anti-Mouse IgG-coupled Qdot605 (#Q11002MP, ThermoFisher Scientific Inc., 1:50000), and nanodiamonds (1:1,000) as fiduciaries and wavelength reference. All incubations and imaging were done in conditioned 1% BSA-supplemented Tyrode solution (in mM: 105 NaCl, 5 KCl, 2 MgCl2, 12 D-glucose, 25 HEPES, pH 7.4). Labelled receptors were imaged for 1,000 consecutive frames at 20 Hz frame rate (Figure 3B). During imaging, a single laser lines at 561 nm was used to excite all three Qdots populations through a quad-band filter set in the lower (spatial) microscope, and a triple laser lines rejection filter (ZET 405/488/561) was added in the upper (spectral) detection path to filter out laser excitation light.

## 3 Results

### 3.1 System Characterization

We first demonstrated the capacity of our method to detect simultaneously five different colors, with single molecule resolution. We imaged five different Qdot populations adsorbed to the surface of a coverslip and excited with a single 488 nm laser (Section 2.3.1). 2,000 frames of the two full chip CCD cameras were acquired at 20 Hz and analyzed by spectrally displaced localization. Figure 2A shows a single frame with the detected Qdots and their assigned wavelength, demonstrating the homogeneity of the wavelength distributions for each quantum dot population. As the 90X effective zoom (60X objective 1.5X zoom) of the upper detection path images a slightly larger field of view and greater depth of field than the lower 100X objective, it maximizes the probability of matching the detected events on the bottom (spatial) channel to a localization on the upper (spectral) channel for their spatially displaced localization. Localization accuracies were then computed for each Qdot population by localizing the same Qdot for 1,000 consecutive frames and Gaussian fitting each localization distribution. Lateral and axial localization accuracies were estimated to ( $\sigma_{xy}=15.0\;nm,\;\sigma_{z}=53.1\;nm)$ for QDots525, ( 15.9 nm, 53.6 nm ) for QDots565, ( 7.5 nm, 12.9 nm ) for QDots605, ( 29.5 nm, 118.7 nm ) for QDots655, and ( 8.6 nm, 21.8 nm ) for QDots565 depending on the brightness of each Qdot population.

### 3.2 3D Multicolor DNA-PAINT Imaging

We then demonstrated the possibility to detect simultaneously up to three organic dyes in a DNA-PAINT imaging strategy. Usually, multi-color DNA-PAINT is made using a sequential approach (Jungmann et al., 2014; Klevanski et al., 2020) but at the expense of a very long acquisition time and numerous washing steps. Here we labelled three cellular structures (microtubules, nucleus envelope (lamin B1) and mitochondria (TOM20)) using three orthogonal docking strands. Then we added the three corresponding imager strands conjugated to three spectrally different fluorophores (ATTO700, ATTO647N and Cy3b) into the imaging media. It enabled us to record simultaneously the 3D nanoscale organization of those three different proteins, increasing by two the overall acquisition time as compared to a sequential acquisition (Figure 2B–i). Super-resolution images were reconstructed from 688,312 (TOM20), 4,501,791 (microtubule) and 636,483 (Lamin) localizations after wavelength assignment and filtering on the goodness of Gaussian fitting (range [0.6, 1]) representing respectively 3.2, 20.9 and 3% of the total localized single molecules that have been assigned to a wavelength by spectrally displaced localization (Figure 2B–ii). Figure 2B–iii shows the distributions of $\sigma_{x}$ and $\sigma_{y}$ single molecule Gaussian fitting parameters for each fluorophore revealing different mean depth of the structure observed.

### 3.3 Multiple 3D QDot Tracking on COS-7 Cells

Next, we performed three color SPT experiments on the biotinylated NCAM membrane protein, labelled with three spectrally different streptavidin labelled Qdots (Qd705, Qd655 and Qd605). We demonstrated our ability to track simultaneously three Qdot populations in 3D at up to 100Hz frame rate (Figure 3A; Supplementary Figure S5). The use of Qdots enables using a single laser line excitation (at 561 nm in our case) for the three species, lowering the overall radiation dose on the sample, and minimizing the phototoxicity. All of the Qdot populations showed similar instantaneous diffusion coefficients distributions (Figure 3A–iii.), validating our capacity to accurately track three different populations simultaneously, in 3D, at high spatial and temporal resolution.

### 3.4 Multiple 3D QDot Tracking of Synaptic Receptors on Living Neurons

Finally, we tracked simultaneously several membrane proteins implicated in synaptic transmission and neurodegenerative disorders on live mixed hippocampal neuron cultures. We probed the same protein (D1R) with secondary antibodies tagged with 2 different quantum dots species (Qdot655 and Qdot705), and the Ephrin B2 receptor with Qdot605 (Figure 3B). Multicolor data were acquired using our spectral microscope and analyzed using spectrally-informed multi-Gaussian fitting and tracking, from which we could extract Qdots trajectories in 3D for each species. Spectrally-informed multi-Gaussian fitting allowed extracting 1.5 more localizations and twice more trajectories, mostly occurring when localized molecules are at a distance below 200 nm (Figure 4C). As expected, both populations of D1R receptors were found on the dendrites, while the Ephrin B2 receptors were mostly localized along the axons (Figure 3B–ii.), validating the specificity of the antibody labeling cocktail. Furthermore, the two sets of D1R receptors showed similar median diffusion coefficients of 0.12 µm·s^−1^ and 0.14 µm·s^−1^, while Ephrin receptors displayed lower median diffusion coefficients of 0.06 µm·s^−1^ (Figure 3B–iii), in agreement with the literature (Mikasova et al., 2012; Ladepeche et al., 2013).

## 4 Discussion

We described a powerful and versatile multidimensional single-molecule localization microscopy workflow. It is composed of a mix of commercially available hardware and software with custom freely available analysis software named PALMTracer. It integrates a spectrally-displaced single molecule localization process and a spectrally-informed multi-Gaussian fitting method allowing to precisely determine the emission wavelength of each localized molecule, and separate overlapping single-molecule signals with high reliability. As spectral determination is performed by collecting and analysing the emitted photons through a second microscope objective, it allows state of the art 3D single molecule localization and tracking to be performed, as well as simultaneous spectral analysis without compromising the spatio-temporal resolution and the field of view. Spectral information also permits to precisely determine the number and 3D coordinates of overlapping, yet spectrally distinct single-molecule emitters.

We intentionally chose to use a low dispersive prism in our spectral detection arm in order to spatially limit the spectral extension onto the camera and allow higher single emitter density to be detected while preserving our capacity to separate several spectrally different populations. With the current configuration, we demonstrated the capability of our approach to detect up to five fluorophores simultaneously, which already represents a substantial biological challenge, more especially for live single molecule applications. This capacity could be further enhanced using fluorescent emitters with sharper spectral emission peak, or high-density based localization approaches and especially recent deep learning-based methods (Nehme et al., 2020; Speiser et al., 2021), whose last implementations outperform standard multi-gaussian methods. Trained for spectrally-displaced localization, it could further enhance the localization and spectral identification precision as well as allow higher detection density, opening new possibilities toward the detection of rare events like protein-protein interactions in real-time. On the other side, certain applications might benefit from higher dispersion capacity that can be easily achieved by replacing the current prism with a more dispersive one into the spectral detection path, in order to observe finer spectral signatures. It could enable, for example, to observe FRET events at the single molecules scale, offering new possibilities to characterize interactions in between bio-molecules. It could also provide a solution to characterize the nano-environment using environment sensitive dyes such a pH- or polar-sensitive dyes for instance (Klymchenko and Mely, 2013; Bongiovanni et al., 2016). It would require no fundamental modification of the current analysis software, but simply a finer description of the detected spectrum. Finally, with further developments to the biological assays and the spectral single particle tracking pipeline, we think our method could be extended in the future to quantify live molecular interactions with nanoscale resolution.

## Data Availability

The raw data supporting the conclusions of this article will be made available by the authors, without undue reservation.

## References

[B1] Babcock H. Sigal Y. M. Zhuang X. (2012). A High-Density 3D Localization Algorithm for Stochastic Optical Reconstruction Microscopy. Opt. Nanoscopy 1, 1–10. 10.1186/2192-2853-1-6 PMC4243665 25431749

[B2] Beghin A. Kechkar A. Butler C. Levet F. Cabillic M. Rossier O. (2017). Localization-based Super-resolution Imaging Meets High-Content Screening. Nat. Methods 14, 1184–1190. 10.1038/nmeth.4486 29083400

[B3] Betzig E. Patterson G. H. Sougrat R. Lindwasser O. W. Olenych S. Bonifacino J. S. (2006). Imaging Intracellular Fluorescent Proteins at Nanometer Resolution. Science 313, 1642–1645. 10.1126/science.1127344 16902090

[B4] Bongiovanni M. N. Godet J. Horrocks M. H. Tosatto L. Carr A. R. Wirthensohn D. C. (2016). Multi-dimensional Super-resolution Imaging Enables Surface Hydrophobicity Mapping. Nat. Commun. 7, 13544. 10.1038/ncomms13544 27929085 PMC5155161

[B5] Chamma I. Levet F. Sibarita J. Sainlos M. Thoumine O. (2016). Nanoscale Organization of Synaptic Adhesion Proteins Revealed by Single- Molecule Localization Microscopy Proteins Revealed by Single-Molecule Localization. Neurophotonics 3, 041810. 10.1117/1.NPh.3.4.041810 27872870 PMC5093229

[B6] Chamma I. Rossier O. Giannone G. Thoumine O. Sainlos M. (2017). Optimized Labeling of Membrane Proteins for Applications to Super-resolution Imaging in Confined Cellular Environments Using Monomeric Streptavidin. Nat. Protoc. 12, 748–763. 10.1038/nprot.2017.010 28277548

[B7] Choquet D. Sainlos M. Sibarita J.-B. (2021). Advanced Imaging and Labelling Methods to Decipher Brain Cell Organization and Function. Nat. Rev. Neurosci. 22, 237. 10.1038/s41583-021-00441-z 33712727

[B8] Cognet L. Leduc C. Lounis B. (2014). Advances in Live-Cell Single-Particle Tracking and Dynamic Super-resolution Imaging. Curr. Opin. Chem. Biol. 20, 78–85. 10.1016/j.cbpa.2014.04.015 24875636

[B9] Compans B. Camus C. Kallergi E. Sposini S. Martineau M. Butler C. (2021). NMDAR-dependent Long-Term Depression Is Associated with Increased Short Term Plasticity through Autophagy Mediated Loss of PSD-95. Nat. Commun. 12, 1–18. 10.1038/s41467-021-23133-9 33990590 PMC8121912

[B10] Cox S. Rosten E. Monypenny J. Jovanovic-Talisman T. Burnette D. T. Lippincott-Schwartz J. (2012). Bayesian Localization Microscopy Reveals Nanoscale Podosome Dynamics. Nat. Methods 9, 195–200. 10.1038/nmeth.1812 PMC3272474 22138825

[B11] Cutler P. J. Malik M. D. Liu S. Byars J. M. Lidke D. S. Lidke K. A. (2013). Multi-Color Quantum Dot Tracking Using a High-Speed Hyperspectral Line-Scanning Microscope. PLoS One 8, e64320. 10.1371/journal.pone.0064320 23717596 PMC3661486

[B12] Dong B. Almassalha L. Urban B. E. Nguyen T. Q. Khuon S. Chew T. L. (2016). Super-resolution Spectroscopic Microscopy via Photon Localization. Nat. Commun. 7, 12290. 10.1038/ncomms12290 27452975 PMC4962472

[B13] El Beheiry M. Dahan M. Vi. S. P. (2013). Representing Single-Particle Localizations in Three Dimensions. Nat. Methods 10, 689–690. 10.1038/nmeth.2566 23900246

[B14] Floderer C. Masson J. B. Boilley E. Georgeault S. Merida P. El Beheiry M. (2018). Single Molecule Localisation Microscopy Reveals How HIV-1 Gag Proteins Sense Membrane Virus Assembly Sites in Living Host CD4 T Cells. Sci. Rep. 8, 1–15. 10.1038/s41598-018-34536-y 30389967 PMC6214999

[B15] Friedman L. J. Chung J. Gelles J. (2006). Viewing Dynamic Assembly of Molecular Complexes by Multi-Wavelength Single-Molecule Fluorescence. Biophys. J. 91, 1023–1031. 10.1529/biophysj.106.084004 16698779 PMC1563747

[B16] Garcia M. Leduc C. Lagardère M. Argento A. Sibarita J. B. Thoumine O. (2015). Two-tiered Coupling between Flowing Actin and Immobilized *N* -cadherin/catenin Complexes in Neuronal Growth Cones. Proc. Natl. Acad. Sci. 112, 201423455. 10.1073/pnas.1423455112 PMC4460488 26038554

[B17] Gustafsson N. Culley S. Ashdown G. Owen D. M. Pereira P. M. Henriques R. (2016). Fast Live-Cell Conventional Fluorophore Nanoscopy with ImageJ through Super-resolution Radial Fluctuations. Nat. Commun. 7, 1–9. 10.1038/ncomms12471 PMC4990649 27514992

[B18] Heilemann M. van de Linde S. Schüttpelz M. Kasper R. Seefeldt B. Mukherjee A. (2008). Subdiffraction-resolution Fluorescence Imaging with Conventional Fluorescent Probes. Angew. Chem. Int. Ed. Engl. 47, 6172–6176. 10.1002/anie.200802376 18646237

[B19] Holden S. J. Uphoff S. Kapanidis A. N. (2011). DAOSTORM: an Algorithm for High- Density Super-resolution Microscopy. Nat. Methods 8, 279–280. 10.1038/nmeth0411-279 21451515

[B20] Huang F. Schwartz S. L. Byars J. M. Lidke K. A. (2011). Simultaneous Multiple-Emitter Fitting for Single Molecule Super-resolution Imaging. Biomed. Opt. Express 2, 1377. 10.1364/BOE.2.001377 21559149 PMC3087594

[B21] Huang T. Phelps C. Wang J. Lin L. J. Bittel A. Scott Z. (2018). Simultaneous Multicolor Single-Molecule Tracking with Single-Laser Excitation via Spectral Imaging. Biophys. J. 114, 301–310. 10.1016/j.bpj.2017.11.013 29401428 PMC5984991

[B22] Izeddin I. Boulanger J. Racine V. Specht C. G. Kechkar A. Nair D. (2012). Wavelet Analysis for Single Molecule Localization Microscopy. Opt. Express 20, 2081–2095. 10.1364/OE.20.002081 22330449

[B23] Jullié D. Stoeber M. Sibarita J. B. Zieger H. L. Bartol T. M. Arttamangkul S. (2020). A Discrete Presynaptic Vesicle Cycle for Neuromodulator Receptors. Neuron 105, 663–677. 10.1016/j.neuron.2019.11.016 31837915 PMC7035187

[B24] Jungmann R. Avendaño M. S. Woehrstein J. B. Dai M. Shih W. M. Yin P. (2014). Multiplexed 3D Cellular Super-resolution Imaging with DNA-PAINT and Exchange-PAINT. Nat. Methods 11, 313–318. 10.1038/nmeth.2835 24487583 PMC4153392

[B25] Kechkar A. Nair D. Heilemann M. Choquet D. Sibarita J.-B. (2013). Real-time Analysis and Visualization for Single-Molecule Based Super-resolution Microscopy. PLoS One 8, e62918. 10.1371/journal.pone.0062918 23646160 PMC3639901

[B26] Klevanski M. Herrmannsdoerfer F. Sass S. Venkataramani V. Heilemann M. Kuner T. (2020). Automated Highly Multiplexed Super-resolution Imaging of Protein Nano-Architecture in Cells and Tissues. Nat. Commun. 11, 1–11. 10.1038/s41467-020-15362-1 32214101 PMC7096454

[B27] Klymchenko A. S. Mely Y. (2013). Fluorescent Environment-Sensitive Dyes as Reporters of Biomolecular Interactions. Prog. Mol. Biol. Translational Sci. 113, 35. 10.1016/b978-0-12-386932-6.00002-8 23244788

[B28] Ladepeche L. Dupuis J. P. Bouchet D. Doudnikoff E. Yang L. Campagne Y. (2013). Single-molecule Imaging of the Functional Crosstalk between Surface NMDA and Dopamine D1 Receptors. Proc. Natl. Acad. Sci. U. S. A. 110, 18005–18010. 10.1073/pnas.1310145110 24127604 PMC3816474

[B29] Lampe A. Haucke V. Sigrist S. J. Heilemann M. Schmoranzer J. (2012). Multi-colour Direct STORM with Red Emitting Carbocyanines. Biol. Cel. 104, 229–237. 10.1111/boc.201100011 22187967

[B30] Lelek M. Melina T. G. Gerti B. Florian S. Juliette G. Suliana M. (2021). Single Molecule Localization Microscopy. Nat. Rev. Methods Prim. 1. 10.1038/s43586-021-00038-x PMC9160414 35663461

[B31] Levet F. Hosy E. Kechkar A. Butler C. Beghin A. Choquet D. (2015). SR-tesseler: a Method to Segment and Quantify Localization-Based Super-resolution Microscopy Data. Nat. Methods 12, 1–9. 10.1038/nmeth.3579 26344046

[B32] Liu X. Longfang Y. Weidong Y. Yiyan F. Lan M. Jiong M. (2019). Spectroscopic Fluorescent Tracking of a Single Molecule in a Live Cell with a Dual-Objective Fluorescent Reflection Microscope. Appl. Phys. Express 12, 112007. 10.7567/1882-0786/ab4b16

[B33] Lundquist P. M. Zhong C. F. Zhao P. Tomaney A. B. Peluso P. S. Dixon J. (2008). Parallel Confocal Detection of Single Molecules in Real Time. Opt. Lett. 33, 1026. 10.1364/ol.33.001026 18451975

[B34] Mikasova L. De Rossi P. Bouchet D. Georges F. Rogemond V. Didelot A. (2012). Disrupted Surface Cross-Talk between NMDA and Ephrin-B2 Receptors in Anti-NMDA Encephalitis. Brain 135, 1606–1621. 10.1093/brain/aws092 22544902

[B35] Moon S. Yan R. Kenny S. J. Shyu Y. Xiang L. Li W. (2017). Spectrally Resolved, Functional Super-resolution Microscopy Reveals Nanoscale Compositional Heterogeneity in Live-Cell Membranes. J. Am. Chem. Soc. 139, 10944–10947. 10.1021/jacs.7b03846 28774176

[B36] Nair D. Hosy E. Petersen J. D. Constals A. Giannone G. Choquet D. (2013). Super-Resolution Imaging Reveals that AMPA Receptors inside Synapses Are Dynamically Organized in Nanodomains Regulated by PSD95. J. Neurosci. 33, 13204–13224. 10.1523/JNEUROSCI.2381-12.2013 23926273 PMC6619720

[B37] Nehme E. Freedman D. Gordon R. Ferdman B. Weiss L. E. Alalouf O. (2020). DeepSTORM3D: Dense 3D Localization Microscopy and PSF Design by Deep Learning. Nat. Methods 17, 734–740. 10.1038/s41592-020-0853-5 32541853 PMC7610486

[B38] Ovesný M. Křížek P. Borkovec J. Švindrych Z. Hagen G. M. Thunder. S. T. O. R. M. (2014). A Comprehensive ImageJ Plug-In for PALM and STORM Data Analysis and Super-resolution Imaging. Bioinformatics 30, 2389–2390. 24771516 10.1093/bioinformatics/btu202 PMC4207427

[B39] Racine V. Hertzog A. Jouanneau J. Salamero J. Kervrann C. Sibarita J. B. (2006). “Multiple-target Tracking of 3D Fluorescent Objects Based on Simulated Annealing,” in 3rd IEEE International Symposium on Biomedical Imaging: Nano to Macro, 2006, Arlington, VA, USA, 6-9 April 2006, 1020–1023. 10.1109/isbi.2006.1625094

[B40] Rossier O. Octeau V. Sibarita J. B. Leduc C. Tessier B. Nair D. (2012). Integrins β 1 and β 3 Exhibit Distinct Dynamic Nanoscale Organizations inside Focal Adhesions. Nat. Cel. Biol. 14, 1057–1067. 10.1038/ncb2588 23023225

[B41] Rust M. J. Bates M. Zhuang X. (2006). Imaging by Stochastic Optical Reconstruction Microscopy ( STORM ). Nat. Methods 3, 793–795. 10.1038/nmeth929 16896339 PMC2700296

[B42] Sage D. Pham T. A. Babcock H. Lukes T. Pengo T. Chao J. (2019). Super-resolution Fight Club: Assessment of 2D and 3D Single-Molecule Localization Microscopy Software. Nat. Methods 16, 387–395. 10.1038/s41592-019-0364-4 30962624 PMC6684258

[B43] Sibarita J.-B. (2014). High-density Single-Particle Tracking: Quantifying Molecule Organization and Dynamics at the Nanoscale. Histochem. Cel. Biol. 141, 587–595. 10.1007/s00418-014-1214-1 24671496

[B44] Song K.-H. Zhang Y. Wang G. Sun C. Zhang H. F. (2019). Three-dimensional Biplane Spectroscopic Single-Molecule Localization Microscopy. Optica 6, 709. 10.1364/optica.6.000709 PMC9854264 36688951

[B45] Speiser A. Müller L. R. Hoess P. Matti U. Obara C. J. Legant W. R. (2021). Deep Learning Enables Fast and Dense Single-Molecule Localization with High Accuracy. Nat. Methods 18, 1082. 10.1038/s41592-021-01236-x 34480155 PMC7611669

[B46] Testa I. Wurm C. A. Medda R. Rothermel E. von Middendorf C. Fölling J. (2010). Multicolor Fluorescence Nanoscopy in Fixed and Living Cells by Exciting Conventional Fluorophores with a Single Wavelength. Biophys. J. 99, 2686–2694. 10.1016/j.bpj.2010.08.012 20959110 PMC2956215

[B47] Thompson R. E. Larson D. R. Webb W. W. (2002). Precise Nanometer Localization Analysis for Individual Fluorescent Probes. Biophys. J. 82, 2775–2783. 10.1016/S0006-3495(02)75618-X 11964263 PMC1302065

[B48] Yan R. Moon S. Kenny S. J. Xu K. (2018). Spectrally Resolved and Functional Super-resolution Microscopy via Ultrahigh-Throughput Single-Molecule Spectroscopy. Acc. Chem. Res. 51, 697–705. 10.1021/acs.accounts.7b00545 29443498

[B49] Zhang Y. Schroeder L. K. Lessard M. D. Kidd P. Chung J. Song Y. (2020). Nanoscale Subcellular Architecture Revealed by Multicolor Three-Dimensional Salvaged Fluorescence Imaging. Nat. Methods 17, 225–231. 10.1038/s41592-019-0676-4 31907447 PMC7028321

[B50] Zhang Z. Kenny S. J. Hauser M. Li W. Xu K. (2015). Ultrahigh-throughput Single-Molecule Spectroscopy and Spectrally Resolved Super-resolution Microscopy. Nat. Methods 15, 935–938. 10.1038/nmeth.3528 26280329

[B51] Zhu L. Zhang W. Elnatan D. Huang B. (2012). Faster STORM Using Compressed Sensing. Nat. Methods 9, 721–723. 10.1038/nmeth.1978 22522657 PMC3477591

