# Supplementary material for: Multi-Dimensional Spectral Single Molecule Localization Microscopy
Source: Front Bioinform. 2022 Mar 4;2:813494. doi: 10.3389/fbinf.2022.813494 (PMC9580959; doi:10.3389/fbinf.2022.813494)
Supplement: Supplementary file 1 [file DataSheet1.pdf]

Multi-Dimensional Spectral Single Molecule Localization Microscopy

Corey Butler[1,2], G Ezequiel Saraceno[1], Adel Kechkar[3], Nathan Bénac[1], Vincent Studer[1], Julien P. Dupuis[1], Laurent Groc[1], Rémi Galland[1*] and Jean-Baptiste Sibarita[1*]

[1]Univ. Bordeaux, CNRS, Interdisciplinary Institute for Neuroscience, IINS, UMR 5297F-33000, F-33000, Bordeaux, France, [2]Imagine Optic, Orsay, France, [3]Ecole Nationale Supérieure de Biotechnologie, Laboratoire de Bioengineering, Constantine, El Khroub, Algeria

Supplementary Figures

Supplementary Figure 1 — 2
Supplementary Figure 2 — 3
Supplementary Figure 3 — 4
Supplementary Figure 4 — 5
Supplementary Figure 5 — 6

Optical Set-up

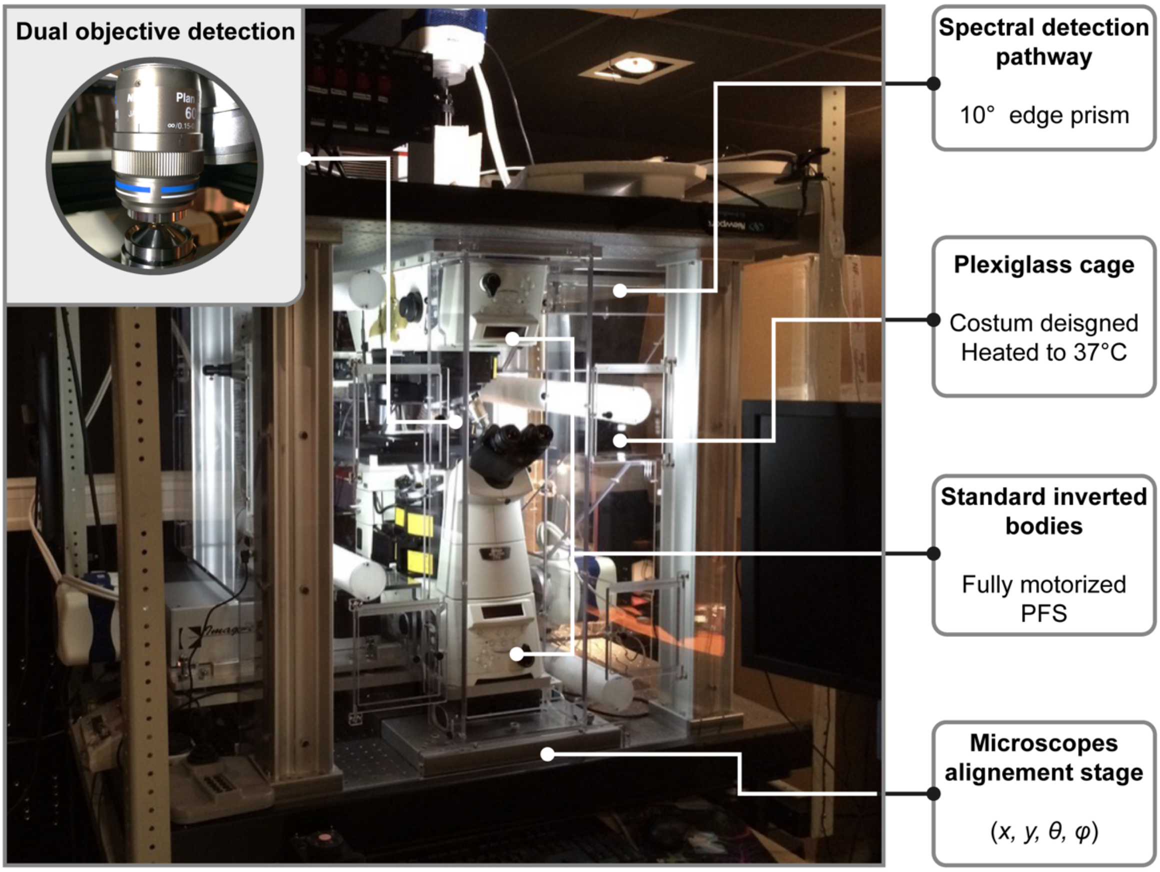

Supplemental Figure 1: Optical set-up description.
Two commercially available fully motorized inverted microscopes, both equipped with a Perfect Focus System (PFS), are mounted on top of each other, and encaged inside a plexiglass cage for temperature control. The lower (spatial) microscope is mounted onto a (x, y, θ, φ) stage for precise microscope alignment with the upper (spectral) microscope. On the upper microscope, a spectral detection path is implemented using a 10° wedge prism. The inset shows the two objectives of each microscope facing each other.

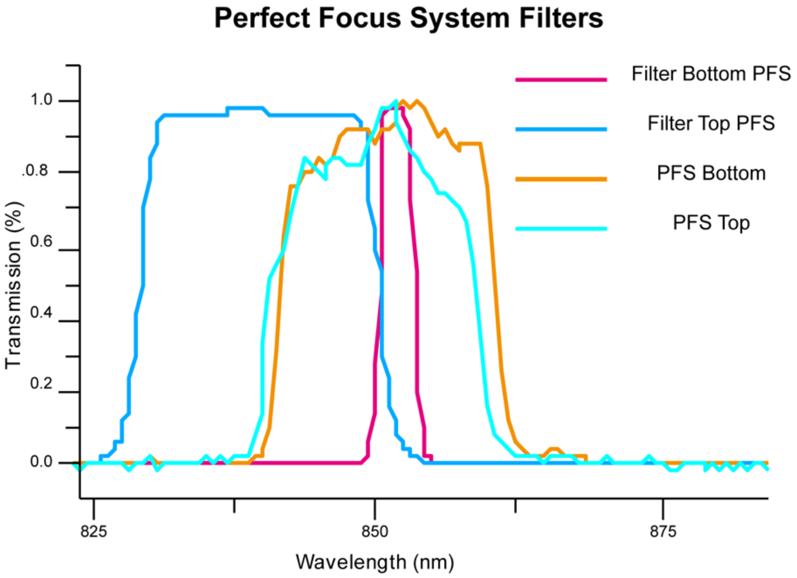

Supplemental Figure 2: Perfect Focus Systems emission and filter sets.
Emission spectra of the two LEDs used on the two Perfect Focus Systems of the lower (orange) and upper (light blue) microscopes. Transmission spectra of the filters added on the upper (blue) and lower (magenta) PFS to avoid interfering when facing each other.

PALMTracer

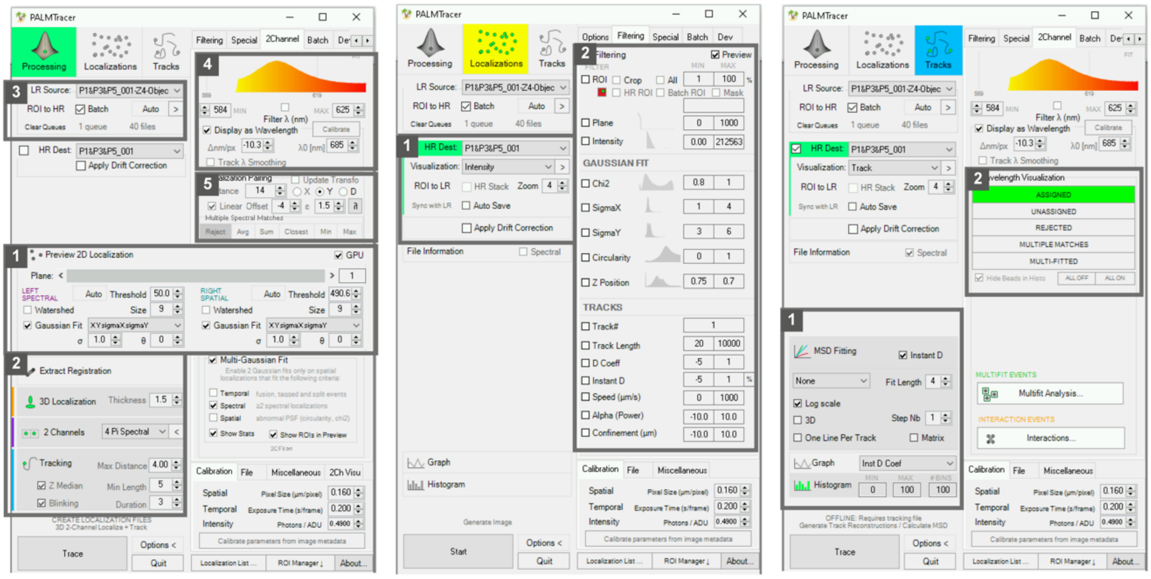

1. Single Molecule Localization
- Separate threshold for the spatial and spectral channels
- Gaussian fitting options

2. Localizaiton options
- 3D localization
- Tracking
- Registration

3. Batch options

4. Wavelength assignment
- Auto-calibration using tetraspeck beads
- Wavelenght filtering option
- Track wavelength averaging

5. Localization pairing
- Pair search zone (Size, offset)
- Multiple pairing options

1. Image reconstruction
- Adjustable zoom
- Representaiton option
- Drift correction

2. Filtering options

1. Trajectories analysis

2. Filtering options

Supplementary Figure 3: PALMTracer interface and main features.
Left: PALMTracer interface used for the 3D localization and the spectrally displaced localization processes as well as for trajectories reconstruction in the spatial and the spectral channels. A batch option enables to analyze several files contained in various folders in a row. Dedicated features enable to calibrate, filter, and define the pairing and spectrally displaced localization options. **Middle**: PALMTracer interface displaying the image reconstruction and many filtering options for the parameters computed during the 3D localization and the spectrally displaced localization processes. **Right**: PALMTracer interface dedicated to the analysis of the trajectories, and the multi-fitting and advanced pairing parameters.

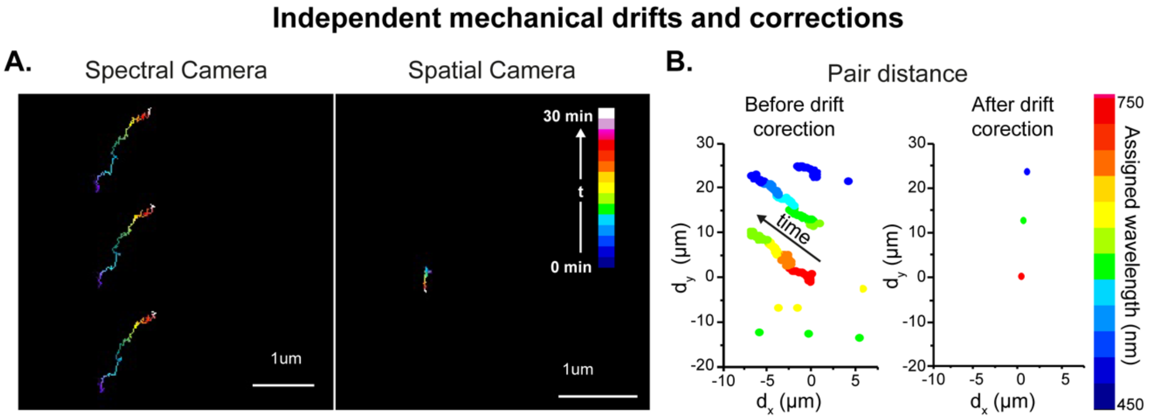

Supplemental Figure 4: Independent drift correction of the spectral and the spatial channels.
(**A**) Example of temporal localizations of a single tetraspeck bead excited at 488, 561 and 640 nm, obtained from a 30 min time-lapse recording at one image every 15 sec, illustrating significant and independent drifts on the spectral (left) and spatial (right) detection channels. On the spectral channel, the three emission peaks of the tetraspeck are localized, while on the spatial channel, only one single molecule event is localized per time point. (**B**) Pair distances computed over the entire time-lapse acquisition before (left) and after (right) drift correction on the two channels independently. Without drift correction, wavelength assignment analysis leads to errors and fluctuations due to drift. Notably, the blue fluorescent dye is only assignable in the first few minutes, after which it extends out of the pair search range. Drift correction restores proper and stable wavelength assignment over the entire acquisition.

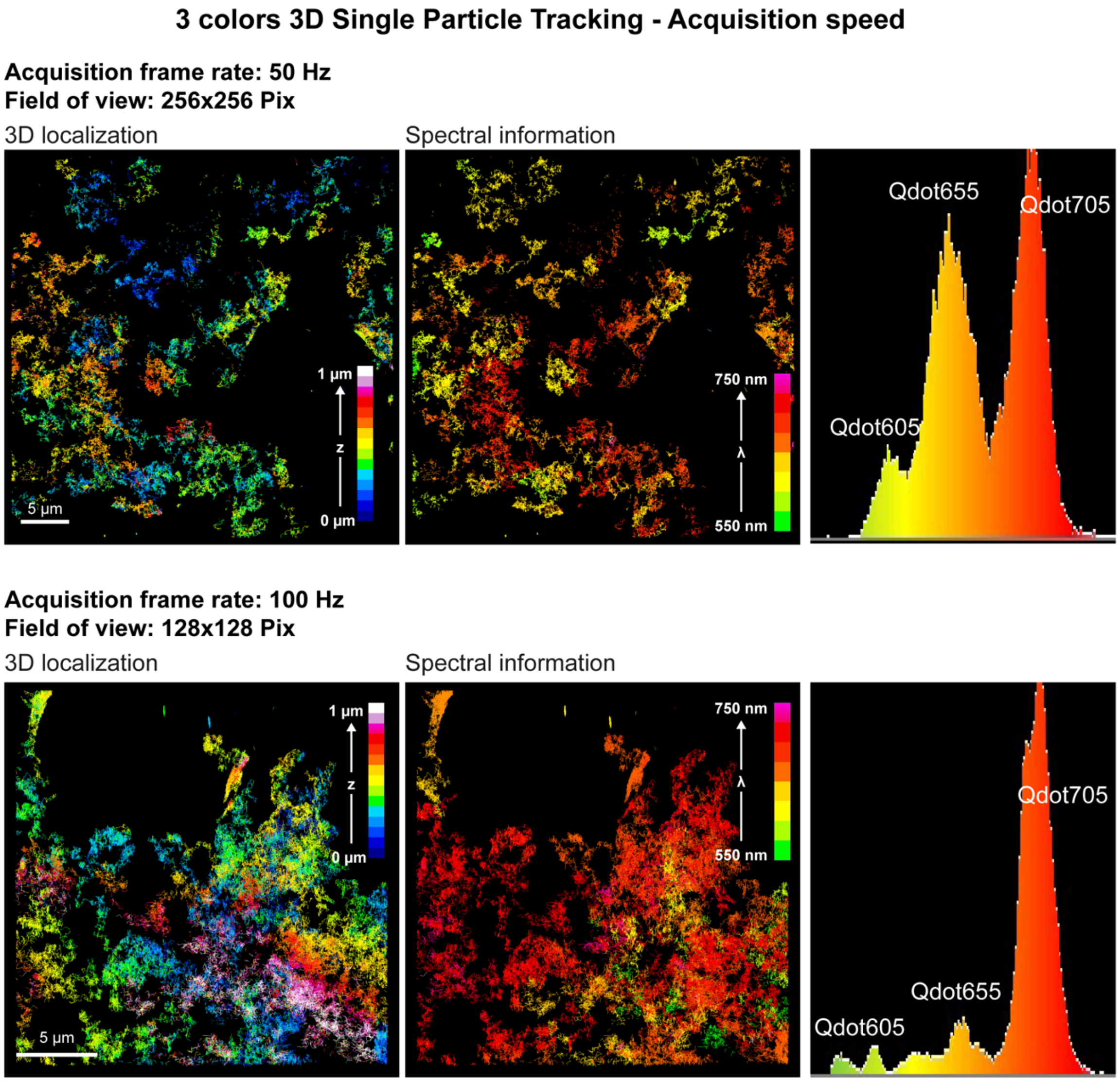

Supplemental Figure 5: Simultaneous multi-color populations tracking at different frame rates. Trajectories reconstructions color coded for the depth (left) or to the assigned wavelength (middle) for the tracking of three spectrally different populations of Qdots (Qdot605, Qdot655 and Qdot705) acquired at frames rates of 50 Hz (top) and 100 Hz (bottom) on two different coverslips having been incubated with slightly different Qdots dilutions due to pipetting accuracy. On the right are represented the histogram of the assigned wavelength for each acquisition, validating the detection of the three different Qdot populations.
